# mHealth Interventions to Support Caregivers of Older Adults: Equity-Focused Systematic Review

**DOI:** 10.2196/33085

**Published:** 2022-07-08

**Authors:** Anna Garnett, Melissa Northwood, Justine Ting, Ruheena Sangrar

**Affiliations:** 1 Arthur Labatt Family School of Nursing Western University London, ON Canada; 2 School of Nursing McMaster University Hamilton, ON Canada; 3 Department of Occupational Science and Occupational Therapy University of Toronto Toronto, ON Canada

**Keywords:** caregivers, older adults, mobile health, social determinants of health, intervention, mobile phone

## Abstract

**Background:**

Informal caregivers, hereafter referred to as caregivers, provide support to older adults so that they can age safely at home. The decision to become a caregiver can be influenced by individual factors, such as personal choice, or societal factors such as social determinants of health, including household income, employment status, and culture-specific gender roles. Over time, caregivers’ health can be negatively affected by their caregiving roles. Although programs exist to support caregivers, the availability and appropriateness of services do not match caregivers’ expressed needs. Research suggests that supportive interventions offered through mobile health (mHealth) technologies have the potential to increase caregivers’ access to supportive services. However, a knowledge gap remains regarding the extent to which social determinants of health are considered in the design, implementation, and evaluation of mHealth interventions intended to support the caregivers of older adults.

**Objective:**

This study aimed to conduct a systematic review to determine how health equity is considered in the design, implementation, and evaluation of mHealth interventions for caregivers of older adults using Cochrane Equity’s PROGRESS-Plus (place of residence, race, ethnicity, culture, language, occupation, gender, religion, education, social capital, socioeconomic status–plus age, disability, and sexual orientation) framework and synthesize evidence of the impacts of the identified caregiver-focused mHealth interventions.

**Methods:**

A systematic review was conducted using 5 databases. Articles published between January 2010 and June 2021 were included if they evaluated or explored the impact of mHealth interventions on the health and well-being of informal caregivers of older adults. mHealth interventions were defined as supportive services, for example, education, that caregivers of older adults accessed via mobile or wireless devices.

**Results:**

In total, 28 articles met the inclusion criteria and were included in the review. The interventions evaluated sought to connect caregivers with services, facilitate caregiving, and promote caregivers’ health and well-being. The PROGRESS-Plus framework factors were mainly considered in the results, discussion, and limitations sections of the included studies. Some PROGRESS-Plus factors such as sexual orientation, religion, and occupation, received little to no consideration across any phase of the intervention design, implementation, or evaluation. Overall, the findings of this review suggest that mHealth interventions were positively received by study participants. Such interventions have the potential to reduce caregiver burden and positively affect caregivers’ physical and mental health while supporting them as caregivers. The study findings highlight the importance of making support available to help facilitate caregivers’ use of mHealth interventions, as well as in the use of appropriate language and text.

**Conclusions:**

The successful uptake and spread of mHealth interventions to support caregivers of older adults will depend on creating opportunities for the inclusive involvement of a broad range of stakeholders at all stages of design, implementation, and evaluation.

## Introduction

### Background

Globally, it is estimated that 101 million older adults require care from a friend or family caregiver (informal caregiver; hereafter referred to as caregiver), with women providing most of the support [1]. The support provided by these informal caregivers is often crucial for enabling older adults to safely remain in their home environment [2-4]. Caregiving support, such as assistance with activities of daily living, attending appointments, and health management, is associated with positive outcomes for both caregivers [5,6] and care recipients [7]. Although caregivers often willingly engage in caring, their role can negatively affect their psychological well-being, particularly when care is provided over a prolonged period [8-10].

### The Social Determinants of Health and Inequities Among Caregivers

The social determinants of health can influence entry into the caregiving role and the subsequent experience of being a caregiver. For example, factors such as being a woman, lower educational attainment, and living in a rural setting can bias caregiving toward individuals who may perceive that they have little agency in their choice to become a caregiver [11]. Furthermore, a greater intensity of caregiving has been identified among caregivers who are female, people of color, and of lower socioeconomic status [12]. These inequities highlight the need for interventions with both scope and accessibility to support caregivers with varied demographic characteristics.

Although some programs and community initiatives are available to support caregivers, the literature suggests that caregivers struggle to access these supportive services [13-15]. Challenges in system navigation, accessing support, geographic location, and scheduling factors can impede the successful use of services [16,17]. Recent research indicates that supportive services provided or augmented through mobile health (mHealth) technologies have the potential to make services more accessible to caregivers [18-20].

### mHealth Interventions as a Potential Solution for Caregiver Support

The term *mobile health* (mHealth) was first coined in 2003 in response to the rapid development and expansion of mobile communication technologies being used within the health care industry [21]. The World Health Organization defines mHealth as a “medical and public health practice supported by mobile devices, such as mobile phones, patient monitoring devices, personal digital assistants, and other wireless devices” [22]. The use of health information technology (computer, internet, and email) to access health records or locate health information on the web has become commonplace among caregivers as a means of informing their caregiving role [23]. Research suggests that mobile apps have the potential to have a greater positive impact on caregivers by providing support, communication, and facilitation of care, thereby reducing the burden and positively affecting caregiver health outcomes [24]. However, to the best of our knowledge, a systematic review of the impact of mHealth support on caregivers of older adults does not currently exist. Furthermore, to date, reviews on standard caregiver interventions suggest that limited work has been conducted to determine the suitability of these interventions for caregivers from backgrounds representing diverse social determinants of health characteristics [25]. Individual characteristics, such as sociodemographic characteristics and the ability to engage with technology, should be considered in the design of mHealth interventions [26].

Therefore, the objectives of this systematic review were to (1) determine how health equity is considered in the design, implementation, and evaluation of mHealth interventions aimed at caregivers of older adults using the Cochrane Equity PROGRESS-Plus (place of residence, race, occupation, gender, religion, education, social capital, socioeconomic status–plus age, disability, and sexual orientation) framework [27] and (2) synthesize the evidence on the impacts of caregiver-focused mHealth interventions, subsequently discussed through a health equity lens.

## Methods

A systematic review was conducted in accordance with the PRISMA (Preferred Reporting Items for Systematic Reviews and Meta-Analyses) statement guidelines [28]. The protocol for this systematic review was registered on PROSPERO (International Prospective Register of Systematic Reviews; CRD42021239584) and is available for electronic access [29].

### Research Questions

The research questions guiding this systematic review were as follows:

- To what extent is health and social equity considered in the design, implementation, and evaluation of mHealth interventions for caregivers of older adults?
- What are the impacts of the examined mHealth interventions on caregivers of older adults based on the following outcomes: caregiver mental and physical health, caregivers’ ability to provide care, usability or feasibility of the mHealth intervention for caregivers, and caregivers’ experiences and perspectives of engaging in an mHealth intervention intended to support them?

### Eligibility Criteria

Eligible articles were available in full text in the English language and were published from 2010 onward to reflect the recent surge in mHealth interventions, concurrent with the rapid increase in mobile device ownership within the past decade [30,31]. This review included both quantitative (experimental, quasi-experimental, and observational studies with or without control or comparison groups) and qualitative study designs, which evaluated or explored the impacts of mHealth interventions aimed at improving the health of, or providing support to, informal caregivers of older adults. Mixed methods studies were also included. mHealth interventions were defined as those that the caregivers of older adults accessed via mobile or wireless devices (including mobile phones, tablets, handheld computers, and PDAs). Interventions not accessed by mobile or wireless devices (eg, interventions applied or accessed by landline telephone as opposed to mobile phone) were excluded, as were mHealth interventions that targeted the recipient of care only or only assessed outcomes focused on the recipient of care. Studies that exclusively included formal caregivers of older adults (eg, nurses and personal support workers) or caregivers of individuals who were not identified as older adults (eg, children, adolescents, young and middle-aged adults, or adults aged <65 years) were also excluded.

Eligible studies were also required to report at least one caregiver-specific outcome or finding, including those relating to (1) caregiver mental and physical health, (2) caregivers’ ability to provide care, (3) usability or feasibility of the mHealth intervention by caregivers, and (4) caregivers’ experiences and perspectives of engaging in mHealth interventions intended to support them. Research protocols, dissertations, reviews, commentaries, and abstracts were also excluded.

### Search Strategy and Study Selection

A systematic search was conducted on five databases: PubMed, PsycINFO (ProQuest), CINAHL, Scopus, and Cochrane Library. An academic librarian was consulted during database search strategy development. Database searches combined a comprehensive suite of similar and related terms for the key domains of *caregivers*, *older adults*, and *mHealth interventions*. Detailed search strategies for each database are provided in Multimedia Appendix 1. The search results were limited by the year of publication from 2010 to February 2021, when the search was initially conducted. The search strategy was repeated in June 2021 to capture newly published articles. Ancestry searches were also conducted using the reference lists of eligible studies, as well as related reviews [19,32-34], to search for additional potential articles for inclusion.

Eligible studies identified from the database and ancestry searches were independently assessed by a group of 4 reviewers (AG, MN, RS, and JT). Each document was reviewed by 2 reviewers (AG, MN, RS, or JT) based on the title and abstract. The full texts of relevant studies were then obtained, and 2 reviewers (AG, MN, RS, or JT) independently examined the full texts of the selected studies to determine the final included articles in accordance with the eligibility criteria outlined previously. Covidence systematic review software (Veritas Health Innovation) was used to organize the search results and facilitate communication between the reviewers. Disagreements were resolved by consensus. In cases where consensus could not be reached, a third reviewer resolved the disagreement.

**Figure 1:**
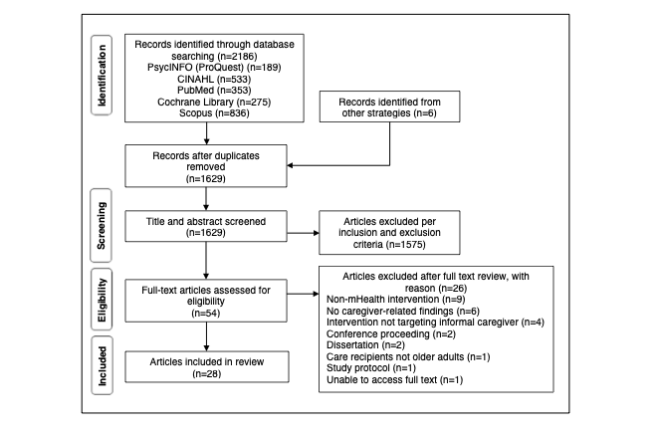
Literature search and study selection process. mHealth: mobile health.

The search strategy yielded an initial 1629 articles for screening of titles and abstracts. On the basis of the initial screening, the full texts of the 3.31% (54/1629) of articles were assessed. Of the 54 articles, 26 (48%) were subsequently excluded after a full-text review. The literature search and study selection processes are shown in Figure 1. A total of 28 articles met the inclusion criteria and were included in the review.

### Data Extraction

The data were extracted using reviewer-designed data extraction forms in Covidence. A total of 2 reviewers independently performed the data extraction. Disagreements were resolved by consensus. In cases where consensus could not be achieved, a third reviewer was consulted.

Data extracted from full-text articles included (1) country of investigation; (2) study design and methods; (3) participant recruitment, demographics, and baseline characteristics; (4) description of the mHealth intervention; and (5) caregiver-specific outcomes or findings. In addition, the review team identified which (if any) social determinants of health and factors contributing to health inequities were addressed by study investigators, as described by the PROGRESS-Plus framework [27,35].

PROGRESS-Plus is a framework developed with evidence from working groups from the Campbell and Cochrane Collaborations, which can be applied to determine whether an equity lens has been used throughout the stages of study design, implementation, and reporting of research [27]. The framework includes the following equity factors: place of residence, race, ethnicity, language, culture, occupation (eg, full-time employment or retirement), gender or sex, religion, education, socioeconomic status, and social capital, as well as age, disability, sexual orientation, features of relationships, and time-dependent relationships (*Plus* factors) [27]. The manner in which investigators addressed these factors within the intervention itself and the study of the intervention was considered in their report of these factors within the following sections: (1) mHealth intervention design, (2) study participant recruitment, (3) study results or findings, and (4) discussion or limitations of the investigation.

### Risk of Bias Assessment

Risk of bias (quality) assessments were performed for each study using standardized critical appraisal tools from the Joanna Briggs Institute Manual for Evidence Synthesis [36]. The Joanna Briggs Institute provides distinct critical appraisal checklists for experimental, quasi-experimental, observational, and qualitative study design. One of the reviewers performed the risk of bias assessments for each study, which was then checked by a second reviewer. Disagreements were resolved by discussion and consensus. No studies were excluded from the review based on quality assessments to achieve a comprehensive understanding of the quality of the available literature exploring the impacts of mHealth interventions for caregivers of older adults. The findings of the quality assessments and the limitations of the included articles are summarized in the results, and the summary scores of the quality assessments are presented in the *Results* section.

### Data Synthesis

A narrative synthesis of findings was pursued because of the range of included mHealth interventions, caregiver characteristics, and caregiver-related outcome measures, as well as the inclusion of both quantitative and qualitative study designs. The narrative synthesis was organized under the following categories: (1) study characteristics; (2) mHealth intervention characteristics; (3) consideration of social determinants and factors contributing to health inequities in mHealth intervention design, participant recruitment, study results or findings, and discussion or limitations; (4) quantitative caregiver-related outcomes; and (5) qualitative caregiver-related findings.

## Results

A total of 28 articles were included in this review. A summary of the included articles is presented in Multimedia Appendix 2 [37-62].

### Characteristics of Included Studies

Among the 28 included studies, 14 (50%) were quantitative [37-48,63,64], 7 (25%) were qualitative [49-55], and 7 (25%) used mixed methods [56-62]. Studies were most frequently conducted in the United States [38,41,45-48,50,53,58,60], the Netherlands [37,55,57,59], the United Kingdom [54,62], and Australia [52,56]. Most studies targeted nonspecific informal caregivers of older adults; however, 25% (7/28) targeted family or spousal caregivers specifically [38,44,51,52,54,60,64]. Approximately 7% (2/28) of studies targeted caregivers who reported being isolated [56] or experiencing caregiving strain [38]. Caregivers most commonly provided care to older adults with dementia or other forms of cognitive impairment [37-39,41-44,46,47,50-56,58-60,62,64]. Other studies recruited caregivers who provided care to older adults with urinary incontinence [63], older veterans who were medically complex [45], and older adults with functional loss or struggling to remain independent at home [49,57,61].

### Risk of Bias Within Included Studies

The full risk of bias assessments for the 28 included studies are presented in Multimedia Appendix 3 [37-62]. The potential for bias within the 11% (3/28) included randomized controlled trials [37,45,46] most commonly stemmed from a lack of blinding of participants and outcome assessors. Potential sources of bias within other quantitative studies include a lack of control groups [60,62,63] and limited consideration of potential confounders [41,56]. Most of the included quantitative studies recruited small convenience samples of caregivers or caregiver–care recipient dyads; for example, recruiting from single clinics [38,39] or from attendees of an Alzheimer’s Association chapter event [50]. Included qualitative studies were most often limited by a lack of clear alignment between philosophical underpinnings, methodology, and research questions or objectives [49-53,55-58,60,62]. Although most studies provided sufficient information to demonstrate a logical flow from the analysis and interpretation of data to the overall conclusions, few studies addressed the potential influence of the researcher on the research (eg, positionality, trustworthiness, and rigor) [54,62]. In addition, only 7% (2/28) of qualitative studies provided information on the location of the researcher’s theoretical approach [53,54]. Although other studies may also have used a theoretical lens or framework to guide their intervention and analysis, they did not report this information.

### mHealth Intervention Characteristics

The included studies’ interventions were web-based or non–web-based applications, interventions, or videoconferencing software, which were delivered via mobile phones, tablets, and handheld computers. The intervention details, including intervention description, hardware, stakeholder input, and comparison groups, are outlined in Table 1.

**Figure 2:**
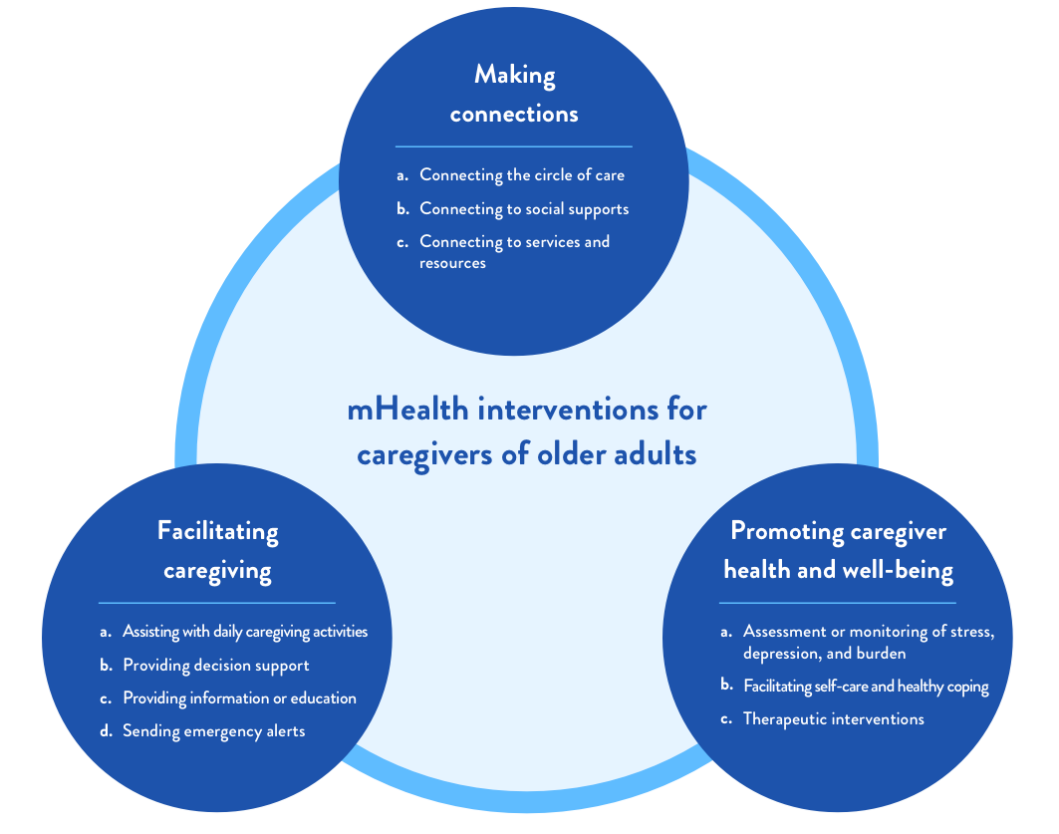
Mobile health (mHealth) interventions for caregivers of older adults.

The aims of these interventions fell under three interrelated categories: making connections, facilitating caregiving, and promoting caregiver health and well-being (Figure 2). The included mHealth interventions facilitated various linkages and connections between caregivers and supportive services, such as (1) connecting the care recipient’s circle of care, including caregivers and health professionals [44,45,48,51,53,55,​^57^,^58^,^61^]; (2) connecting the caregiver to existing social support or facilitating new connections to peer support [40,43,46,56,59]; and (3) connecting the caregiver to services and resources for both themselves and the recipient of care [37,43,47,50,51,53,58].

**Table 1:** Details of mobile health interventions of included studies.

Study		Intervention description	Hardware provided	Stakeholder input described	Comparator intervention (as applicable)	Study quality appraisal scores^a^
**Quantitative studies—randomized controlled trials**						
	Beentjes et al [37]	FindMyApps, a web-based selection tool and learning training program to help caregivers find user-friendly apps	Yes; tablet	No	Caregiver controls received a tablet but no FindMyApps training or access; received a list of links to websites with apps for people with dementia or mild cognitive impairment	8/13
	Hastings et al [45]	Video-enhanced care management: a 14-week care management intervention that included 3 monthly video calls with nurses via a secure internet-based web-based meeting room	Yes; tablet	No	One group received the intervention (video); the comparator group received telephone-based care management	5/13
	Kales et al [46]	WeCareAdvisor, a web-based tool for family caregivers, which guided them through a clinical reasoning process to identify, monitor, and manage behaviors while addressing their motivation, self-efficacy, and problem-solving skills	Yes; tablet	No	Waitlist for the tool; this group received the tool 1 month later	8/13
**Quantitative studies—quasi-experimental**						
	Davis et al [63]	TelePrompt, a tablet-based, prompted voiding and educational intervention to support caregivers of older adults with urinary incontinence	Yes; tablet	No	No comparison group; the study was described by authors as a quasi-experimental, single-group pre-post design	6/9
	Lai et al [44]	Telehealth delivered via videoconferencing platforms (apps) aimed at minimizing the possible negative impact of social distancing measures made necessary by the COVID-19 pandemic	No	No	Received a weekly care service via telephone covering information relevant to caregiving; did not receive the intervention of weekly health services delivered through video communication apps	7/9
	Park et al [64]	Comprehensive Mobile Application Program, a tool providing real-time support to families caring for patients with dementia by helping family caregivers manage behavior and psychological symptoms	No	No	Comparator intervention was a handbook that contained the same information as the mobile app	5/9
	Watcharasarnsap et al [42]	A mobile app system based on the reminiscence therapy framework; the app was developed to promote the relationship between caregivers and people with dementia and better the mental well-being of both parties	No	No	Control group did not use the intervention (no intervention)	9/9
**Quantitative s** **tudies** **—** **other** **(ie, noncomparative)**						
	Callan et al [38]	A self-administered cognitive training intervention using an adaptive, paced serial attention task, targeting the dorsolateral prefrontal cortex, which is implicated in regulating emotions, anxiety, and stress	Yes; handheld computer	No	N/A^b^	6/10
	Davis et al [43]	An e-mobile multimedia app for community-based dementia caregiver support, designed to offer reassurance, information, and services to caregivers and facilitate the implementation of other interventions by nurses and therapists	Yes; mobile phone	No	N/A	1/10
	Ptomey et al [47]	A remotely delivered exercise intervention to increase moderate physical activity in caregivers	Yes; tablet	No	N/A	4/10
	Quinn et al [48]	A mobile app designed to improve engagement of the patient-informal caregiver team; the mobile web-based app allowed older adult users to record social and health information and share this information with their caregivers	No	No	N/A	4/10
	Lai et al [39]	A simple smartphone app for people with mild cognitive impairment and their family caregivers living in the community; the app supported communication with friends and family, navigation, and serving as a memory prompt and emergency alert system	Yes; mobile phone	No	N/A	6/10
	Salin and Laaksonen [40]	A multicomponent intervention, including live broadcasts related to caregiver self-care exercises, informational videos, and videoconferencing web-based meetings to connect informal caregivers	Yes; tablet	Yes	N/A	2/10
	Sourbeer et al [41]	A preliminary tablet app developed for the Behavioral and Environmental Sensing and Intervention for Dementia Caregiver Empowerment; the goal of this app is to support the early detection of signs of agitation, allowing caregivers to intervene early	Yes; tablet	No	N/A	2/11
**Mixed methods studies**						
	Banbury et al [56]	A telehealth peer-support program for isolated caregivers of people with dementia via group videoconferencing	Yes; not specified	No	N/A	3/8 and 3/10
	Breebaart and van Groenou [57]	A groupware app for digital network communication to promote collaboration among informal and formal caregivers in a mixed care network of home-dwelling older adults	Yes; not specified	No	N/A	1/10 and 3/10
	Brown et al [58]	CareHeroes, an app providing caregivers with a platform for bidirectional sharing of observations and knowledge with providers about care recipients and, in so doing, provide them with information and support for caregiving activities	No	Yes	N/A	4/10 and 3/10
	Dam et al [59]	Inlife, a web-based social support platform for caregivers of individuals with dementia aiming to enhance positive interaction, involvement, and social support	No	No	Control group did not receive the intervention (waiting list)	4/10 and 7/10
	Sikder et al [60]	A mobile app intervention delivering mentalizing imagery therapy (a guided imagery and mindfulness intervention) for family caregivers	No	No	N/A	5/9 and 3/10
	Stutzel et al [61]	A mobile phone app, The Mobile System for Elderly Monitoring, which aimed to support caregivers in monitoring care recipients with functional loss and to improve support for caregivers’ communication with the health team	Yes; mobile phone	Yes	N/A	5/10 and 7/10
	Tyack et al [62]	An art-based app intervention delivered via a touch screen tablet displaying art images aiming to stimulate and benefit the well-being of caregivers and care recipients with dementia	Yes; tablet	Yes	N/A	6/9 and 8/10
**Qualitative studies**						
	Garvelink et al [49]	A decision support website to inform caregivers about ways of staying independent at home for as long as possible, called Supporting Seniors and Caregivers to Stay Mobile at Home	No	No	N/A	3/10
	Hughes et al [50]	A tablet app with multiple components, including games and a stress questionnaire for caregivers	No	Yes	N/A	5/10
	Killin et al [51]	The Digital Support Platform, an internet-based, postdiagnostic support tool for families of individuals who had recently received a diagnosis of dementia	Yes; tablet	No	N/A	6/10
	Rathnayake et al [52]	Mobile health apps used for health information seeking	No	No	N/A	7/10
	Ruggiano et al [53]	CareIT, a multifunctional smartphone and web-based app designed to meet the education and support needs of caregivers; the app allowed caregivers to self-assess for depression and burden and linked caregivers to resources	Yes; mobile phone	Yes	N/A	5/10
	Ryan et al [54]	InspireD—Individual Specific Reminiscence in Dementia, a personalized reminiscence program for family carers and people living with dementia	Yes; tablet	Yes	N/A	10/10
	Span et al [55]	The DecideGuide, an interactive web tool that helps informal caregivers, people with dementia, and case managers make shared decisions	Yes; tablet	Yes	N/A	5/10

^a^Complete quality appraisal tools and scores are presented in Multimedia Appendix 3.

^b^N/A: not applicable.

mHealth interventions included in the review also facilitated caregiving by (1) assisting with daily caregiving activities (eg, digital calendars to organize appointments, providing reminders for medication administration, helping caregivers manage care recipient behaviors, and tracking information related to the care recipient) [39,41,46,48,51,53,57,59,61,63,64], (2) providing support for decisions related to care [46,49,55,58], (3) providing information or education (eg, regarding the care recipient’s condition) [40,43-46,48,49,51-53,56,58,63,64], and (4) sending emergency alerts to the caregiver or to the care team if needed [39,61].

Finally, the mHealth interventions represented in the review promoted caregiver health and well-being by (1) monitoring or assessing caregiver stress, depression, and burden to facilitate early detection and intervention before reaching crisis levels [41,50,53,58,61]; (2) promoting self-care and healthy coping behaviors (eg, encouraging physical activity or suggesting evidence-based coping strategies for care recipient behaviors) [40,43,47,63,64]; and (3) providing therapeutic interventions (eg, art-based interventions [62], reminiscence therapy [42,54], cognitive training therapy [38], and mentalizing imagery therapy [60]).

### Consideration of Factors That Influence Health Inequities

**Figure 3:**
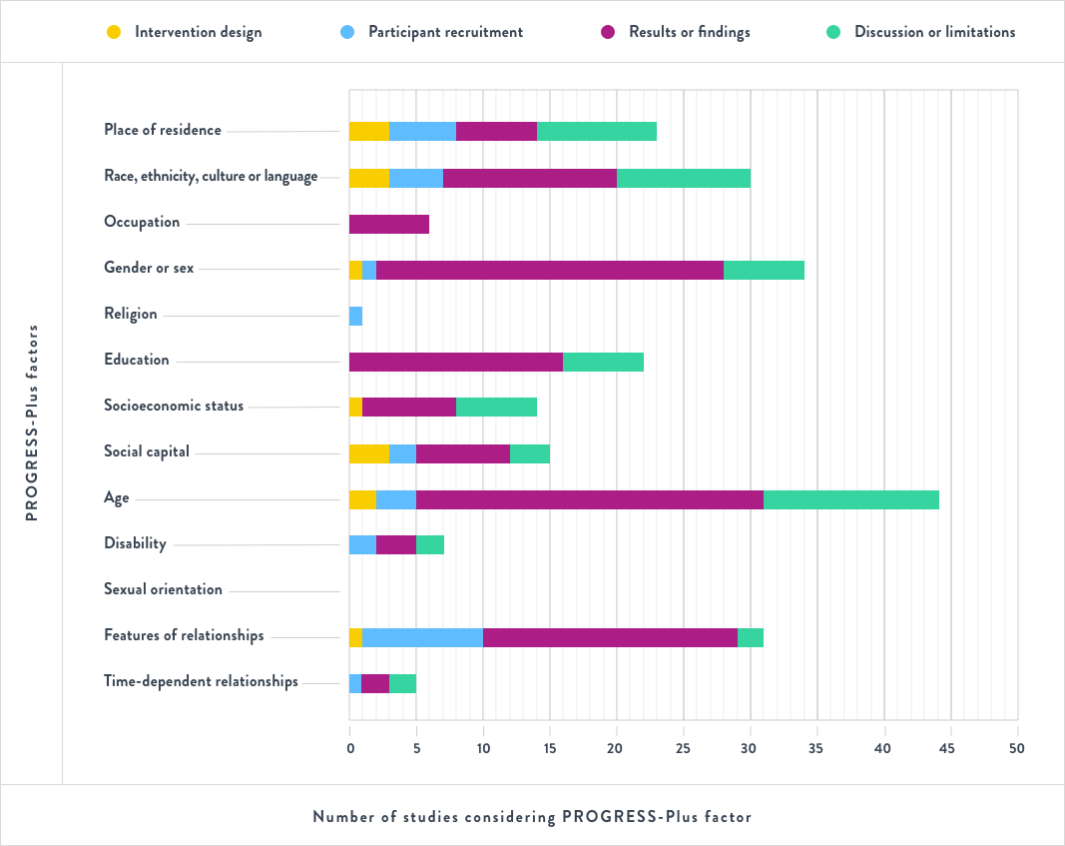
Consideration of place of residence, race, occupation, gender, religion, education, social capital, socioeconomic status–plus age, disability, and sexual orientation (PROGRESS-Plus) factors in included studies.

Figure 3 provides a visual summary of the number of studies that included or considered the factors listed in the PROGRESS-Plus framework in their report on (1) the design of their mHealth intervention, (2) participant recruitment, (3) study results or findings, and (4) study discussion or limitations.

#### Reporting of PROGRESS-Plus Factors in Intervention Design

When describing the design of their interventions, 36% (10/28) of studies provided considerations for ≥1 PROGRESS-Plus factor [37,40,41,46,48,49,56,59,61,63]. Approximately 11% (3/28) of studies considered the place of residence in their recruitment approaches as their interventions were designed specifically for geographically isolated caregivers [40,56,61]. Approximately 11% (3/28) considered languages through the provision of alternative language options in the mobile app, readability (ie, lay language), and accessibility options such as larger font or less text [37,46,49]. Approximately 11% (3/28) described social capital as an element of the intervention itself (eg, intervention aimed at providing a platform to organize and access social support) [56,59,63]. Approximately 7% (2/28) described considerations for caregivers’ age in the design of their interventions by improving readability, comprehensibility, and clarity of the language used in the intervention; providing caregivers with assistance in completing web-based forms; and integrating opportunities for regular check-ins to support mHealth tool use [41,46]. One of the studies considered gender or sex, as the intervention was tailored to address the unique needs of caregivers of different genders [46]. Another study considered socioeconomic status by deliberately selecting inexpensive mobile apps and devices [61]. Features of relationships between caregivers and care recipients were considered in the study design such that the mHealth intervention was a collaborative tool whereby older adults and their caregivers worked together on their health management [48]. None of the studies mentioned considering participants’ occupation, religion, education, disability, sexual orientation, or time-dependent relationships when describing the design of their mHealth interventions.

#### Reporting of PROGRESS-Plus Factors in Participant Recruitment

At the participant recruitment stage, 57% (16/28) of studies considered ≥1 PROGRESS-Plus factor [38,40,42,44-46,49,51-53,56-60,64]. Approximately 32% (9/28) considered features of relationships (eg, living situation) [38,40,44,46,51,52,58,60,64]. Approximately 18% (5/28) of studies considered place of residence in participant recruitment (eg, recruiting participants dwelling in rural areas) [40,42,53,56,57]. Approximately 14% (4/28) of studies reported that they used specific recruitment strategies to help ensure that various races, ethnicities, cultures, and languages were represented in their study samples (eg, recruiting from minority populations) [46,49,53,58]. Approximately 11% (3/28) of studies considered age (eg, recruiting older caregivers) [38,45,60]. Approximately 7% (2/28) of studies considered social capital (eg, recruiting caregivers with an existing social support network) [56,59] and 7% (2/28) considered disability (eg, excluding caregivers with sensory impairment) [38,46]. One of the studies considered time-dependent relationships (eg, excluding dyads where the care recipient was awaiting imminent institutional placement) [46], and another considered gender or sex [59] at the stage of participant recruitment. No studies mentioned occupation, religion, education, socioeconomic status, or sexual orientation during participant recruitment.

#### Reporting of PROGRESS-Plus Factors in Results or Findings

All but 1 study [54] described ≥1 PROGRESS-Plus factor within their results or findings. These factors were typically reported as part of the sample demographics. The key demographic characteristics of the caregivers in the included studies are presented in Table 2. The most commonly reported PROGRESS-Plus factors within the included articles’ results or findings were age and gender or sex [37-50,52,53,55-64]; features of relationships [37,39,40,42,43,45,46,48,49,51-53, 55-59,61,63]; education [37-39,44,46-50,52,55,56,58,61,63,64]; and race, ethnicity, culture, and language [38,41,43,45-49,53,58,60,62,63]. Other factors reported in the results or findings included socioeconomic status [38,44,48,53,61,63,64], social capital [48,55-57,59,61,64], place of residence [40,49,53,56,62,64], and occupation [50,52,56,61,63,64]. A small number of studies reported on caregivers’ disabilities [49,61,63], time-dependent relationships (eg, participants’ housing situation) [49,58], and religion [64]. No studies reported on sexual orientation in their results or findings.

**Table 2:** Demographic characteristics of caregiver participants of included studies.

Study and country	Sample size	Age (years)	Sex, n (%)	Education, n (%)	Ethnicity, n (%)
Banbury et al [56], Australia	69	Mean 62.6 (SD 13.54)	50 (72.5) female 19 (27.5) male	6 (8.7) did not complete high school 6 (8.7) completed high school 17 (24.6) had technical and further education or trade 24 (34.8) attended university 16 (23.2) had postgraduate qualifications	Not reported
Beentjes et al [37], Netherlands	59	Experimental group mean 65.61 (SD 10.196); control group mean 68.03 (SD 11.675)	38 (64.4) female 21 (35.6) male	12 (20.3) had secondary education (vocational) 8 (13.6) had secondary education (academic) 11 (18.6) had further education (vocational) 20 (33.9) had higher education (vocational) 8 (13.6) had higher education (academic)	Not reported
Breebaart and van Groenou [57], Netherlands	7	1 (14.3%) middle-aged, 1 (14.3%) aged between 60 and 65, and 5 (71.4%) aged ≥70	3 (42.9) female 3 (42.9) male 1 (14.3) not specified	4 (57.1) had low education 2 (28.6) had average education 1 (14.3) did not specify	Not reported
Brown et al [58], United States	11	Mean 56.6 (SD 13.6)	9 (81.8) female 2 (18.2) male	Not reported	3 (27.3%) White 7 (63.6%) African American 1 (9.1%) Hispanic 1 (9.1) other
Callan et al [38], United States	27	Mean 74.61 (SD 6.52)	22 (81.5) female 5 (18.5) male	11 (40.7) had middle school to technical school education 14 (51.9) had some college to college graduate education 2 (7.4) had some postgraduate to postgraduate degree	26 (96.3) White
Dam et al [59], Netherlands	10	Range 49-71	6 (60) female 4 (40 male)	Not reported	Not reported
Davis et al [43], United States	4	Mean 52	4 (100) female	Not reported	Not reported
Davis et al [63], United States	3	Range 54-85	3 (100) female	2 (66.7) attended college 1 (33.3) had a master’s degree	3 (100) White
Garvelink et al [49], Canada and France	10	Mean 56.9 (SD 14)	6 (60) female 4 (40) male	10 (100) had a university degree	Not reported
Hastings et al [45], United States	40	Mean 64.7 (SD 10.8)	40 (100) female	Not reported	11 (27.5) Black
Hughes et al [50], United States	10	Mean 60 (range 48-76)	10 (100) female	10 (100) had high school education 9 (90) had higher education	Not reported
Kales et al [46], United States	57	Mean 65.9 (SD 14.0)	43 (75.4) female 14 (24.6) male	48 (84.2) had greater than high school education 9 (15.8) had high school or GEDa	36 (63.2) White 18 (31.6) African American 3 (5.3) other
Killin et al [51] [51], Scotland	10	Not reported	Not reported	Not reported	Not reported
Lai et al [44], Hong Kong, China	60	Experimental group mean 72.43 (SD 0.80, range 66-82); control group mean 71.83 (SD 0.80, range 66-82)	35 (58.3) female 25 (41.7) male	Experimental group: 7.90 (SD 0.25, range 5-11) years of education Control group: 7.04 (SD 0.31, range 5-9) years of education	Not reported
Lai et al [39], Germany	24	Mean 62.4 y (SD 16.0, range 31-83)	9 (37.5) female 15 (62.5) male	11 (45.8) had >12 years of education	Not reported
Park et al [64], South Korea	24	Experimental group mean 54.50 (SD 3.71); control group mean 61.00 (SD 6.42)	14 (58.3) female 10 (41.7) male	15 (62.5) were high school graduates or below 9 (37.5) were college graduates or above	Not reported
Ptomey et al [47], United States	9	Mean 67	3 (33.3) female 6 (66.7) male	3 (33.3) had high school diploma or GED 6 (67.6) attended postgraduate classes	8 (88.9) White 1 (11.1) Black
Quinn et al [48], United States	12	Mean 54.8 (SD 13.3)	11 (91.7) female 1 (8.3) male	6 (50) had a business or some college degree or graduate degree 6 (50) graduated school	6 (50) Black 6 (50) White
Rathnayake et al [52], Australia	10	8 (80%) aged <65; 2 (20%) aged ≥65	9 (90) female 1 (10) male	5 (50) had high school education and below 5 (50) had above high school education	Not reported
Ruggiano et al [53], United States	36	Mean 65.7 (range 42-89)	26 (72.2) female 10 (27.8) male	Not reported	13 (36.1) non-Hispanic White 23 (63.9) African American
Ryan et al [54], United Kingdom	17	Mean 69.1 (SD 15.1, range 31-91)	13 (76.5) female 4 (23.5) male	Not reported	Not reported
Salin and Laaksonen [40], Finland	20	Range 61-88	15 (75) female 5 (25) male	Not reported	Not reported
Sikder et al [60], United States	17	Mean 66.52 (SD 9.61)	12 (70.6) female 5 (29.4) male	Not reported	17 (100) White
Sourbeer et al [41], United States	46	42 (91.3%) aged >60; 4 (8.7%) aged <60	38 (82.6) female 8 (17.4) male	Not reported	39 (84.8) White 6 (13.0) African American 1 (2.2) Hispanic
Span et al [55], Netherlands	12	Mean 54.3 (range 19-86)	7 (58.3) female 5 (41.7) male	1 (8.3) had low education 4 (33.3) had medium education 6 (50) had high education 1 (8.2) did not specify	Not reported
Stutzel et al [61], Brazil	38	Mean 61 (SD 10.75)	32 (84.2) female 6 (15.8) male	21 (55.3) had ≤12 years of education 17 (44.7) had >12 years of education	Not reported
Tyack et al [62], United Kingdom	12	Mean 66 (range 48-77)	10 (83.3) female 2 (16.7) male	Not reported	12 (100) White
Watcharasarnsap et al [42], Thailand	60	8 (13.3%) aged between 18 and 27, 19 (31.7%) aged between 28 and 37, 15 (25%) aged between 38 and 47, 10 (16.7%) aged between 48 and 57, and 8 (13.3%) aged ≥58	31 (51.7) female 29 (48.3) male	Not reported	Not reported

^a^GED: General Educational Development.

#### Reporting of PROGRESS-Plus Factors in Discussion or Limitations

Approximately 79% (22/28) of studies considered ≥1 PROGRESS-Plus factor in the discussion or limitations sections of their studies [37,38,40,41,43,44,47-56,58-63]. The most frequently discussed PROGRESS-Plus factors in the included articles’ discussion or limitations were age [37,38,40,41,43,48,50,51,53,54,60,62,63], such as challenges faced by older caregivers in using mobile devices; race, ethnicity, culture, and language [40,41,43,47,49,52,53,55,58,60], such as a lack of diversity of the study sample; and place of residence [40,44,47,49,51,53,55,56,61], such as challenges related to the lack of access to stable internet in rural locations. Other PROGRESS-Plus factors described in the study discussions or limitations were gender or sex [38,41,52,54,55,63], education [37,38,49,52,56,63], and socioeconomic status [44,47,48,52,53,63]. To a lesser extent, caregivers’ social capital [48,56,59], disabilities [38,49], features of relationships (eg, nature of relationship between caregiver and care recipient) [54,55], and time-dependent relationships (eg, the impact of COVID-19 on the amount of time caregivers could spend visiting the care recipient) [37,49] were also discussed. No studies considered occupation, religion, or sexual orientation in their discussion or limitations sections.

### Quantitative Caregiver Outcomes

#### Outcomes Relating to Caregiving

Approximately 21% (6/28) of studies assessed the impact of mHealth interventions on outcomes related to caregivers’ capabilities or experiences in providing care. These outcomes included caregivers’ self-efficacy [44,63], sense of competence [37] and confidence [46] in their caregiving role, knowledge related to the care recipient’s condition [63], positive care experience [37], and caregiver burden [43,44,46,63,64]. Although some studies found that caregiving self-efficacy and knowledge improved after the implementation of an mHealth intervention [44,63], other studies observed no difference after the intervention in caregivers’ sense of competence [37], confidence [46], or positive caregiving experience [37].

Approximately 14% (4/28) of studies using the Zarit Burden Inventory [65] found that mHealth interventions led to improvements in caregiver burden [43,44,46,64]. However, one of the studies, which specifically assessed caregiver burden related to the management of urinary incontinence, found that burden was similar before and after the mHealth intervention [63]; however, study investigators noted that the intervention did not worsen caregiver burden [63].

#### Outcomes Relating to Caregivers’ Health and Well-being

Approximately 39% (11/28) of studies assessed the impact of mHealth interventions on various aspects of caregivers’ health and well-being [37,40,42-44,46,47,60-62,64]. Impacts on caregivers’ mental and psychological health status were assessed in 25% (7/28) of studies [42,44,46,60-62,64], with generally positive results. Specifically, mental health status [44], psychological well-being [42], depression [46,60], mood [60], distress [46], and fatigue [64] were each noted to have improved after the implementation of an mHealth intervention. For example, the implementation of the WeCareAdvisor tool, designed to provide caregivers with peer navigation, information, and daily messaging, led to significant improvement in self-reported distress (−6.08, SD 6.31 points; P<.001) [46]. In this study, those in the control group demonstrated a significant decrease in their confidence in caregiving (−6.40, SD 10.30; P=.002) [46]. Conversely, a study that assessed caregiver stress by testing cortisol levels in saliva in a pretest-posttest design found no differences after the use of an mHealth intervention designed to manage the behavioral and psychological symptoms of dementia [64]. Caregivers’ self-appraised happiness was also unchanged after the intervention in one of the studies [62].

Approximately 11% (3/28) of studies assessed outcomes related to caregivers’ physical health and well-being [44,47,64]. Caregivers self-reported improvements in their general physical health status following the use of an mHealth intervention to support the well-being and community living of older adults and their spousal caregiver dyads [44]. Ptomey et al [47], who implemented an mHealth app to encourage exercise, observed that caregivers’ weekly moderate physical activity increased by 49 minutes (30% increase) per week over the 12-week intervention period, whereas light physical activity increased by 11.6 minutes (3% increase) per week. However, Park et al [64] found no difference in caregivers’ sleep quality after the implementation of a supportive mHealth app.

Approximately 14% (4/28) of studies used caregivers’ quality of life as an outcome measure for their respective interventions, with mixed findings. Ptomey et al [47] found nonsignificant trends toward improvement in quality of life after the implementation of an mHealth intervention. Beentjes et al [37] and Tyack et al [62] found no significant changes in quality of life following their interventions aimed at supporting caregivers in finding user-friendly apps and viewing art to encourage therapeutic reminiscence, respectively. Salin and Laaksonen [40] observed that some aspects of quality of life, in fact, worsened, albeit mildly (breathing, sexual activity, vitality, depression, and usual activities). One of the studies assessed the impact of an mHealth intervention on caregivers’ social engagement and found high positive responses using the Kaye Gain Through Involvement Scale [66], suggesting that the gains in well-being experienced while using the mHealth intervention may be applicable when tested in a larger sample [43]. However, the study investigators noted that their sample was meant only for determining intervention efficacy and warranted testing with a larger sample [43].

#### Outcomes Related to Usability, Feasibility, and Acceptability of mHealth Interventions

Half of the reviewed studies assessed outcomes related to the usability, acceptability, or feasibility of mHealth interventions for caregivers of older adults [38-41,45,47,48,57-63].

Approximately 32% (9/28) of studies measured the usability or ease of use of mHealth interventions by caregivers [40,41,45,47,48,58,59,61,63]. Approximately 14% (4/28) of articles used the System Usability Scale [67] to do so; usability scores varied across studies, ranging from marginally acceptable [45], moderate [48], and good to excellent [61]. Only 4% (1/28) of studies compared the system usability scores across 2 phases of their mHealth app intervention. Sourbeer et al [41] found that usability did not significantly improve in a subsequent version of their mHealth app updated in response to participant feedback. The remaining 18% (5/28) of studies assessed caregivers’ ease of use or perceived user-friendliness of the mHealth intervention using descriptive statistics or averaged Likert scale scores. These studies generally reported positive results, suggesting that caregivers believed the interventions were easy or very easy to use [40,47,58,59,63].

Approximately 21% (6/28) of studies examined caregivers’ satisfaction or positive feelings toward the intervention [39,40,47,48,58-61]. Most reported that caregivers were generally satisfied with the mHealth intervention, perceived the intervention as relevant and useful to their caregiving activities, and felt positive about their experiences with the intervention [39,40,47,48,58-61]. However, greater technical difficulties were reported in a study of participants who lived rurally and reported lower levels of satisfaction [40].

Approximately 29% (8/28) of studies explored the feasibility of an mHealth intervention by measuring the regularity, frequency, and extent of its use by caregivers over the intervention period [38,57-60,62]. Use varied across the included studies, and investigators did not consistently establish expectations of use for their participants nor defined what constituted adequate use of the intervention. Tyack et al [62] reported that the participants used their app at least five times during the intervention period, as suggested by the study investigators. Callan et al [38] found that 22 out of 27 (81.5%) caregivers used the mHealth intervention regularly (as defined by the study investigators as at least 3 weeks out of the 4-week intervention period). Baseline caregiver stress, worry, and sleep quality did not adversely affect the use of the mHealth intervention, and caregivers with the highest self-reported stress and worry reported the highest levels of mHealth intervention use [38]. Sikder et al [60] reported that over half of their 17 study participants accessed ≥75% of the informational documents in their mHealth app. The remaining 11% (3/28) of studies reported varying frequencies or hours of use per week during the intervention period [57-59]; however, these studies did not comment on whether these frequencies constituted low, medium, or high use of their mHealth interventions.

Approximately 11% (3/28) of studies assessed feasibility by measuring the intervention attendance and retention of caregivers during the intervention period [40,45,47]. The attendance rates for caregivers varied from 72% (13.7/19) [40] to 97.1% (34/35) [45]. Ptomey et al [47] and Hastings et al [45] reported similar figures (7/9, 78% dyads, and 31/40, 78% dyads, respectively) for the caregiver–care recipient dyads completing their interventions.

Other feasibility measures used by the reviewed studies included the extent to which caregivers followed or adhered to the mHealth intervention [38,63]. Callan et al [38] reported that caregivers’ continued engagement in a cognitive training mHealth intervention program was evidenced by improvements in their ability to perform cognitive training tasks. Davis et al [63] reported that caregivers were capable of learning and implementing the prompted toileting strategies to support care recipients with the help of an mHealth intervention, as evidenced by a reduction in care recipient wetness in 2 out of 3 participant dyads.

### Qualitative Caregiver Findings

#### Overview

Of the 28 studies, 7 (25%) qualitative studies and 7 (25%) mixed methods studies presented findings relating to caregivers’ experiences of engaging in mHealth interventions [49-62]. These qualitative findings included (1) positive impacts of caregivers’ experiences with mHealth interventions, (2) challenging aspects of caregivers’ experiences with mHealth interventions, (3) barriers to caregivers’ engagement with mHealth interventions, and (4) caregivers’ suggestions to improve mHealth interventions.

#### Positive Experiences With mHealth Interventions

Most studies highlighted promising findings related to the positive impacts of caregivers’ experiences with mHealth interventions. Participants across the included studies found mHealth interventions to be helpful, user-friendly, and easy to understand [49,50,54,55]. mHealth interventions were perceived to help caregivers connect with the care team and provide care for their loved ones [53,55,57,60,61]. The information provided through mHealth interventions was described as relevant to addressing participants’ educational needs [49,52]. Caregivers also valued the role of mHealth interventions in detecting their stress levels [50] and facilitating timely connections to a diverse range of professional services and social support [49,52,54,56,62]. Participants in the included studies reported benefits to their emotional and cognitive well-being [60,62] and described reappraising and feeling closer to the care recipient [54,62]. The mobile delivery of the interventions also contributed to feelings of safety and security, as caregivers could participate from their homes [54,56]. Although some participants initially felt a lack of confidence in using technology, caregivers in 7% (2/28) of studies reported becoming more engaged and comfortable over time by integrating the mHealth intervention into their lives [54,57].

#### Challenging Experiences With mHealth Interventions

Several studies described the negative aspects of caregivers’ experiences with using mHealth interventions, although these were often reported as being applicable to only a minority of participants. Approximately 11% (3/28) of studies indicated that some participants felt that the mHealth intervention was too complex or difficult to understand [49,51,60]. In another study, participants felt that the intervention included questions that were overly obtrusive or confronting; for example, participants were not always comfortable answering questions they perceived as challenging [55]. Some studies highlighted caregivers’ concerns regarding the potentially detrimental impacts of mHealth interventions; for example, interventions that facilitated reminiscence could trigger painful memories and lower mood [54,62]. Hughes et al [50] further described caregivers’ concerns regarding the diversion of their time and attention toward the mHealth intervention and away from the care recipient. One of the studies highlighted the preference of some participants for in-person interventions, citing physical contact as an important element of care (eg, hugging), which was not possible in a digital environment [56].

#### Barriers to Caregivers’ Engagement With mHealth Interventions

Caregivers relayed frustration with the usability of mHealth interventions, including difficulties navigating the intervention on their mobile devices [49,50,62]. Challenges included print that was too small [49,50], screens that were overly sensitive or had too much glare [62], and language that was too complex [49]. Several studies highlighted a lack of familiarity or experience with technology as a key barrier to the use of mHealth interventions, particularly for older caregivers [51-53,55]. The busy schedules of caregivers for older adults were also identified as a barrier to regular mHealth intervention use, particularly if caregivers were often pulled away from their devices by care recipients or if they were experiencing health issues themselves [50,52,58,60].

In other cases, participants felt that the intervention’s content was not relevant to their immediate needs [49,51] or lacked realism (eg, lack of ethnic diversity among actors portraying caregivers in the mHealth intervention) and up-to-date links to relevant resources [49]. Other barriers included the prohibitive cost of mobile devices and internet or data plans [52] and the availability of a stable internet connection in rural regions [56].

#### Caregivers’ Perspectives Regarding Next Steps

Qualitative findings frequently incorporated participants’ suggestions to make mHealth interventions more user-friendly and accessible to caregivers. Suggestions included simplifying the intervention’s interface or instructions, enlarging text and images, and including subtitles on video resources for individuals with hearing impairment [49,52,61,62]. Participants voiced the need for ongoing technical support, particularly for caregivers who were unfamiliar with using mobile devices [51,56].

The participants also made suggestions to develop more relevant and up-to-date content for mHealth interventions. Several studies highlighted the need to embed local and national services for caregiver support, including interventionists and respite care [58-60]. For interventions that targeted the caregiver–care recipient dyad, participants highlighted the need for more information specifically related to their own health, such as healthy coping [49,52,58,61]. Participants also called for greater emphasis on topics that caregivers often find difficult, including information about deciding to move to a care home, managing activities of daily living and aggressive behaviors, and resources for individuals experiencing abuse [49,52,58].

Other findings suggested to improve mHealth engagement among caregivers included greater ethnic diversity portrayed within the mHealth intervention [49], establishing a reward system to encourage regular use [50], and creating a component for the care recipient to be included when the caregiver uses the mHealth intervention [50].

## Discussion

### Principal Findings

This systematic review examined how health and social equity are considered in the design, implementation, and evaluation of mHealth interventions developed for caregivers of older adults using the PROGRESS-Plus framework. The interventions described in the included studies were designed to create linkages between caregivers and external supports, streamline and optimize caregiving activities, and encourage a focus on caregiver health and well-being. As such, evidence on the impacts of caregiver-focused mHealth interventions was synthesized across a range of outcomes.

The findings indicate that health and social factors are not consistently taken into consideration when designing research studies (ie, used to develop and guide recruitment and intervention design). Furthermore, participant characteristics are most often only reported within study results when summarizing participant characteristics or when identifying limits to the generalizability of the findings. However, this review highlights how mHealth interventions are well-positioned to improve caregivers’ self-efficacy and knowledge, their perceived mental and physical wellness, and their relationships with care recipients. The usability and acceptability of mHealth interventions were characterized by ease of use, ease of navigating technical challenges, and relevance of intervention content to the caregivers’ individual roles and context.

### Consideration of PROGRESS-Plus Factors in Studies on mHealth Interventions for Caregivers of Older Adults

#### Overview

Most studies in this systematic review on mHealth interventions for caregivers of older adults considered some PROGRESS-Plus factors, particularly when describing their study samples. However, such demographic reporting reflects standardized reporting practices of participant composition rather than deliberate and targeted approaches to recruiting caregivers across sociodemographic characteristics to determine whether an intervention is suitable for a diversity of participants. The factors described in the following sections were considered critical in the intervention design.

#### Gender Sex or Sexual Orientation

Importantly, few studies considered actively recruiting caregivers of different self-reported genders or considered the relevance of gender in intervention design or implementation. Research suggests that biological and gender differences affect health across a range of parameters such as risk, disease incidence, and the need for health services [27]. Furthermore, sexual orientation was, in fact, eclipsed across all studies, particularly when many studies focused on caregiver health and well-being, which includes the relationship they have with care recipients. Recent evidence indicates that sexual and gender minority caregivers, such as those identifying as queer and transgender, report higher depressive symptoms (78%) than the overall population of caregivers of people with dementia (34%) [68]. This finding highlights the importance of diversifying samples across genders and sexual orientations to reliably assess and address caregivers’ mental health. The importance of considering the intersections among gender, sexual orientation, and other sociodemographic factors was also highlighted in the survey of a cross-sectional sample of members of the National Alliance for Caregiving. Caregivers who identified as lesbian, gay, bisexual, and transgender were more likely to be racially and ethnically diverse and represent lower socioeconomic classes than those who did not [69].

#### Education

Education, although frequently reported in demographics, was also rarely considered as an important factor in informing intervention design and recruitment. Women with lower education are more likely to assume caregiving roles than those who have had additional educational opportunities [11]. Lower literacy levels among caregivers can affect their ability to navigate the health system and locate appropriate support for themselves and their care recipient [70], factors that can directly influence the design and usability of mHealth interventions. For example, lower literacy can affect comprehension of text-based content in mHealth apps, the ability to correctly enter spelled words in search functions, and the ability to navigate app menus [71]. The importance of designing mHealth interventions that account for varying levels of educational background is underscored by the association of literacy with health and digital literacy [72].

The findings of the included studies suggest that experience with technology can be a key barrier to the use of mHealth interventions, particularly among older caregivers [51-53,55]. A survey of a broad age range of caregivers suggests that younger caregivers (aged <50 years) are more than twice as likely than older caregivers to be receptive to using mHealth apps to support them in their caregiving roles [24]. For older adults, trust in technology as it relates to privacy and access to information can be an important factor in the use of mHealth interventions, especially given the heterogeneity of this population [24,73]. These findings suggest that exploring barriers and facilitators, as identified by the included qualitative studies, aimed at educating older adults on how to use mHealth interventions is essential to facilitate perceived trust, comfort, and usability of technology. Thus, beyond education as a social determinant of health, wide disparities exist across caregivers in comfort with using various technologies, such as tablets, iPads, and mobile phones [73].

#### Socioeconomic Status

Socioeconomic status was minimally considered in the intervention design and was most often addressed when describing sample characteristics. Multiple studies reported providing participants with devices to support the use of mHealth apps [37-41,43,45-47,51,53-57,61-63]. In some cases, participants were allowed to keep the devices; however, especially in those instances in which they were not, the feasibility of such interventions for caregivers across income levels needs to be explored.

Some interventions were designed to facilitate communication access to health professionals and other individuals (eg, support groups), highlighting the need for access to a reliable internet connection. This lack of access may be due in part to financial constraints, as a survey of caregivers in the United States found that cost was a commonly reported barrier to the use of technology [74]. Furthermore, older adults living on fixed incomes may be reticent to spend money on devices they do not value or find overly complicated [75]. Importantly, older caregivers tend to have fewer technological devices than their younger peers, and these technologies are often used for communication purposes rather than health management purposes [18]. Although most caregivers report valuing technology, those that use it for health-related activities tend to use it for targeted caregiving activities such as medication tracking or safety [18]. Therefore, additional support or education may be required to increase caregiver uptake of mHealth interventions as a tool for addressing broader caregiver needs such as communication with health teams or liaising with other caregivers. Computers and smartphones are increasingly being owned by people with higher income and education, and the provision of caregiver support through mHealth apps could increase inequalities if economic resources are not considered in the design and implementation of these interventions [71].

#### Culture, Language, and Race

The nature of caregiver–care recipient relationships can be an important factor in the design of mHealth tools, particularly when it comes to cultural expectations of family members, gender roles, and other caregiver demographics. The included studies had samples primarily made up of women, validating the literature that suggests women are most likely to provide caregiving support, corroborating cultural norms across a range of identities [76]. However, these studies did not address how intersecting identities (eg, culture, gender, race and ethnicity, and socioeconomic status) might shape expectations and responsibilities within a caregiving role [11,12,68]. Research suggests that culture strongly affects caregiving but that cultural influences on the caregiver role must be understood within the context of race and gender socialization [77]. For example, individualistic or Western notions of strategies to address caregiver burdens, such as spending time alone or sharing caregiving responsibilities with friends or family, might not resonate with caregivers from other cultures, particularly those with a strong sense of filial responsibility or immigrant caregivers without local support [78]. Furthermore, mHealth apps not provided in caregivers’ first languages decrease accessibility and would require careful translation and cultural adaptation to remain meaningful [79]. The impact of these factors on caregiver-specific outcomes, such as caregiving self-efficacy, health and well-being, and technology usability, is yet to be explored. Intersecting identities are increasingly important to consider when tailoring web-based caregiver interventions to participants’ individual needs [19].

### mHealth Interventions Developed for Caregivers of Older Adults

#### Impacts of mHealth Interventions on Caregiver Health and Wellness

Studies evaluating mobile technology interventions aiming to promote caregivers’ perceived mental and psychological health reported benefits to their emotional and cognitive well-being [60,62]. Some of these interventions, such as the videoconferencing platform developed by Lai et al [44], were designed in lieu of in-person community services, following shelter-in-place orders during the COVID-19 pandemic. Connecting caregivers to professional and peer support using web-based technologies has been shown to improve mental health outcomes and can help caregivers overcome common access-related barriers related to PROGRESS-Plus factors, such as geographical and time constraints or community mobility limitations related to physical or mental health [18-20]. However, findings from the included studies suggest that caregivers still require opportunities for in-person interaction (eg, hands-on training from a health care provider to successfully use external support systems), suggesting that the impact of hybrid models of interventions to improve caregiver health and wellness is not well understood [20]. Furthermore, a review of these interventions using the PROGRESS-Plus factors suggests that, although caregivers stand to benefit from mHealth interventions and many older adults report being comfortable with smartphone use, uptake may continue to be constrained if support is not provided to help caregivers learn and familiarize themselves with mHealth apps at the outset [80]. Hybrid approaches have the potential to increase caregiver self-efficacy, as opposed to overwhelming caregivers with new tools and technology, which warrants further research.

#### Supporting the Caregiver Role Through mHealth Interventions

Caregivers’ ability to perform their roles was a key focus of the examined mHealth interventions and outcomes of interest within the included studies. Although some interventions focused on creating external structures that facilitated responsibilities of providing care (eg, medication alarms, and checklists), the use of these tools had the potential to complicate caregiving responsibilities. For example, in one case, caregivers described that the increased *screen time* to engage in the intervention was taking away from the time they had to complete other caregiving tasks [58]. The impact of such detrimental experiences, as they relate to, for example, PROGRESS-Plus factors of gender-informed cultural caregiving roles, features of relationships, or caregiver disability, is not well understood. Wasilewski et al [34] found that caregivers’ decline in web-based intervention use may be attributed to a malalignment with their specific needs and capabilities across the caregiving trajectory. In such cases, it is important for those recommending mHealth interventions to caregivers to consider whether a particular intervention itself might increase the caregiver burden [81]. Furthermore, research suggests that if older adults perceive an mHealth app to be beneficial to their health and well-being, their likelihood of ongoing and increased engagement with other apps increases [82]. Individualized tailoring of mHealth apps and providing the necessary access and universal design can foster equitable uptake and increase the potential benefits of mHealth interventions.

#### Usability, Feasibility, and Acceptability of mHealth Interventions

Overall, caregivers in the included studies were generally comfortable using mHealth interventions and reported positive impacts on their caregiving role [49,50,54,55]. However, findings such as the prohibitive costs associated with mobile devices and internet and data plans, in combination with the quality of internet provision to those living in rural settings, highlight the importance of equitable service provision across the PROGRESS-Plus factors [52,56]. The findings of this review also showed that 64% (18/28) of studies [37-41,43,45-47,51,53-57,61-63] provided participants with the devices required to engage in the interventions, suggesting that the economic feasibility of these interventions needs to be better understood.

Technical features such as app use data may provide valuable insights into the frequency and applicability of interventions to caregiver needs and their unique lifestyles. Furthermore, researchers have been urged to include older adults and their caregivers in the design and development of mobile app technologies [48]. However, a minority of the studies included in this review described stakeholder input as a component of their intervention design or implementation [40,50,53-55,58,61]. Co-design approaches present important opportunities for engaging diverse populations to help ensure that mHealth interventions are inclusive and accessible.

### Implications

Moving forward, an important reminder is that social determinants of health should be consciously considered in all aspects of mHealth intervention design and implementation to avoid perpetuating inequities experienced by historically and currently systemically disadvantaged caregivers of older adults living with chronic conditions [25,83]. Purposeful efforts to include a diverse range of participants in research, such as evidence-based recruitment strategies, can help redress these potential inequities and inform the development of more inclusive interventions [84,85]. The PROGRESS-Plus framework is an appropriate tool to help ensure that a health and social equity lens is applied in research design and reporting, the use of which should be widely endorsed [27,86].

This review highlights the need for high-quality mHealth studies. Particular attention must be paid to improving the design of mHealth interventions and ensuring equality in access and adoption of mHealth interventions [71]. Participatory action approaches to research, such as co-design, are ideal for ensuring that mHealth interventions meet the needs of diverse caregivers. Furthermore, inclusive design principles can be used in more traditional research methodologies to ensure that mHealth interventions do not amplify health disparities. This could be achieved by accommodating low literacy by including audio narration and visual depictions or by directing funding to increase access to human resource infrastructures (eg, technical support) that promote mHealth interventions in remote or low-income regions [71].

### Strengths and Limitations

The studies included in this systematic review represent the diversity of mHealth interventions that have been conceptualized and created to address caregiver needs. Unfortunately, many studies were found to be poorly designed and executed. Although half of the included studies assessed usability, feasibility, and acceptability of mHealth interventions, which are all important aspects of technology use, many of these used qualitative approaches and lacked overall methodological rigor. Given the variety of mHealth apps, technological devices, and implementation protocols, equivalent comparisons could not be made across studies. A small number of studies were identified evaluating the impact of caregiver-focused interventions on caregiver-specific outcomes, limiting the ability to make conclusive recommendations to guide practice. Encouragingly, some of the included quantitative studies that used valid and reliable standardized tools thoroughly described their approach to statistical analysis and generally addressed fidelity of intervention delivery.

In this review, multiple steps were taken to achieve methodological rigor. The review was conceptualized and designed using an equity framework and the best evidence on interventions for caregivers of older adults. The search strategy was developed in consultation with a health research librarian, and database searches, screening, data extraction, and risk of bias evaluations were conducted in duplicate, with a strong agreement between reviewers. The review protocol was also made publicly available a priori and was adhered to without any deviations. In addition, the PRISMA and PRISMA-Equity guidelines guided each phase of this study [28,87].

Inevitably, this study has some limitations. Although these searches were conducted by health and rehabilitation investigators across 3 large academic institutions in the Global North, these institutions use similar health research databases and search algorithms, which can affect future reproducibility (ie, replicating searches in different institutions with different journal accesses). The identification of potentially eligible literature from the Global South, other disciplines beyond health research (eg, technology literature databases), or those that are categorized in other ways (eg, gray literature) is another limitation of this review. However, this study highlights that research on mHealth interventions for caregivers of older adults primarily occurs within applied health settings. As such, future reviews should examine non–peer-reviewed evidence such as reports and program evaluations produced by the government and health authorities that trial mHealth interventions.

This study could have been further strengthened by involving additional team members, such as administrators of clinical settings who would implement mHealth interventions and, most importantly, caregivers of older adults themselves. By selecting the PROGRESS-Plus framework as a theoretical guide, this study did not examine the included interventions and investigations in light of compounding factors that disadvantaged caregivers (eg, impact of the intervention on older women living in rural settings) or capture other health and social factors beyond the framework (eg, access to health insurance). However, using the framework as an approach to name and identify how key individual factors have been considered in intervention design and evaluation, this study has set the stage for future investigations that examine the confluence of multiple social determinants of health.

### Conclusions

mHealth supports are well-positioned to support caregivers of older adults by providing them with information, communication, and assistance in their caregiving role. However, access, uptake, and the ability to benefit from this technology can be affected by the social determinants of health and inequities among caregivers. This systematic review of mHealth interventions to support caregivers of older adults suggests that these tools are well-received by caregivers and have the potential to support caregivers across a variety of parameters by facilitating education, communication, and a sense of security for caregivers. The social determinants of health and equity factors are not widely considered in the design and implementation of mHealth interventions, although these parameters are frequently collected for demographic reporting. Recognizing that there are many challenges in designing and implementing mHealth interventions that are equitable, going forward, it will be important to strive for greater inclusion of the social determinants of health at all stages of mHealth development and implementation if there is to be widespread successful uptake of this supportive technology.

## Appendix

Multimedia Appendix 1 Search strategies for all databases.

Multimedia Appendix 2 Summary table of included studies.

Multimedia Appendix 3 Risk of bias assessments.

## Abbreviations

mHealth: mobile health
PRISMA: Preferred Reporting Items for Systematic Reviews and Meta-Analyses
PROGRESS-Plus: place of residence, race, occupation, gender, religion, education, social capital, socioeconomic status–plus age, disability, and sexual orientation
PROSPERO: International Prospective Register of Systematic Reviews
