# Supplementary figures and images for: mHealth Interventions to Support Caregivers of Older Adults: Equity-Focused Systematic Review
Source: JMIR Aging. 2022 Jul 8;5(3):e33085. doi: 10.2196/33085 (PMC9308083; doi:10.2196/33085)

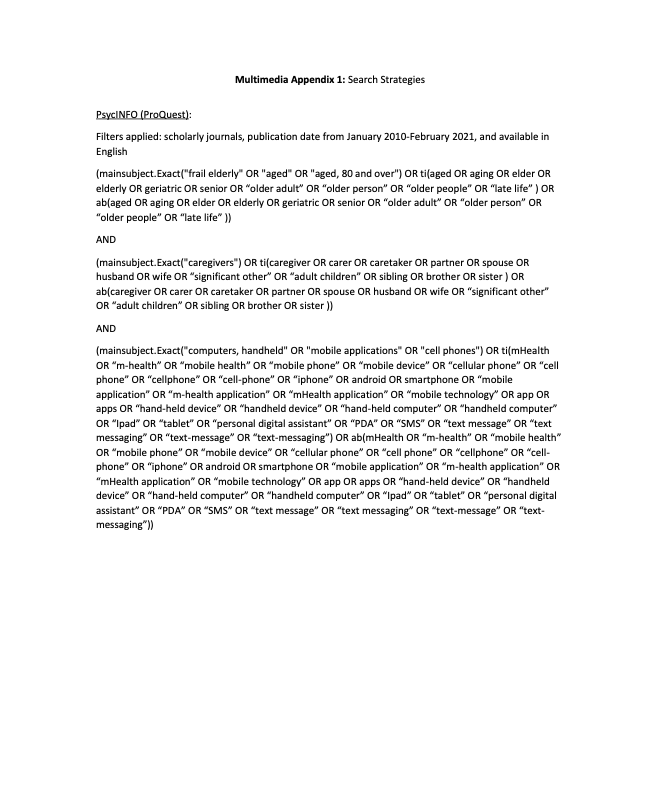

Supplement: Multimedia Appendix 1 [file aging_v5i3e33085_app1.png]

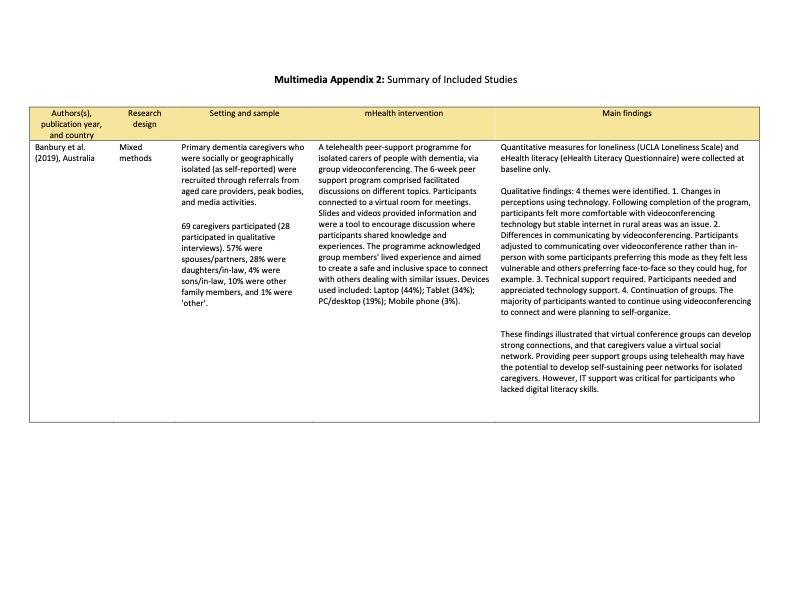

Supplement: Multimedia Appendix 2 [file aging_v5i3e33085_app2.png]

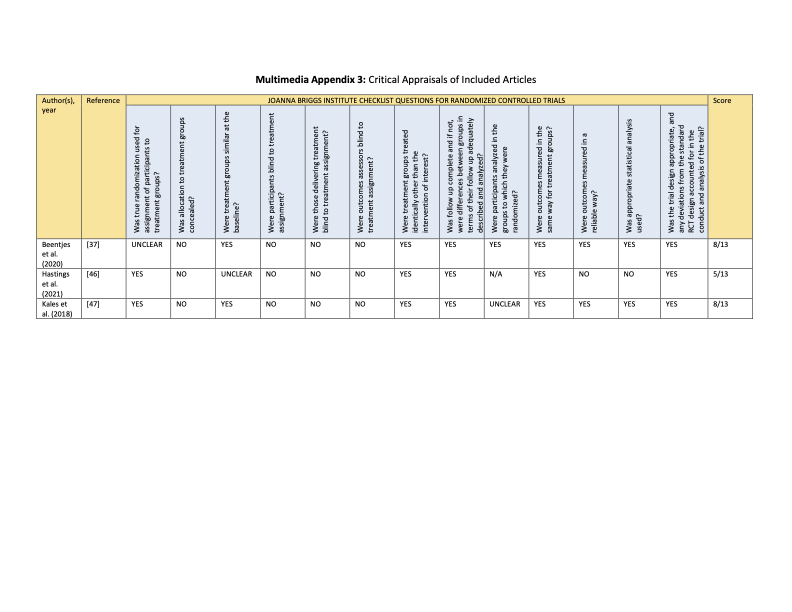

Supplement: Multimedia Appendix 3 [file aging_v5i3e33085_app3.png]
